# The MET Oncogene as a Therapeutical Target in Cancer Invasive Growth

**DOI:** 10.3389/fphar.2012.00164

**Published:** 2012-09-11

**Authors:** Paolo Luraghi, Florian Schelter, Achim Krüger, Carla Boccaccio

**Affiliations:** ^1^Division of Experimental Clinical Molecular Oncology, IRCC – Institute for Cancer Research and Treatment, University of Turin Medical SchoolCandiolo, Italy; ^2^Klinikum rechts der Isar der Technischen Universität München, Institut für Experimentelle Onkologie und TherapieforschungMünchen, Germany

**Keywords:** MET oncogene, invasion, metastasis, microenvironment, targeted therapy, proteases, antibody, small molecule kinase inhibitors

## Abstract

The MET proto-oncogene, encoding the tyrosine kinase receptor for Hepatocyte Growth Factor (HGF) regulates invasive growth, a genetic program that associates control of cell proliferation with invasion of the extracellular matrix and protection from apoptosis. Physiologically, invasive growth takes place during embryonic development, and, in post-natal life, in wound healing and regeneration of several tissues. The MET oncogene is overexpressed and/or genetically mutated in many tumors, thereby sustaining pathological invasive growth, a prerequisite for metastasis. MET is the subject of intense research as a target for small molecule kinase inhibitors and, together with its ligand HGF, for inhibitory antibodies. The tight interplay of MET with the protease network has unveiled mechanisms to be exploited to achieve effective inhibition of invasive growth.

## The MET Tyrosine Kinase and the Invasive Growth Program

**Figure 1:**
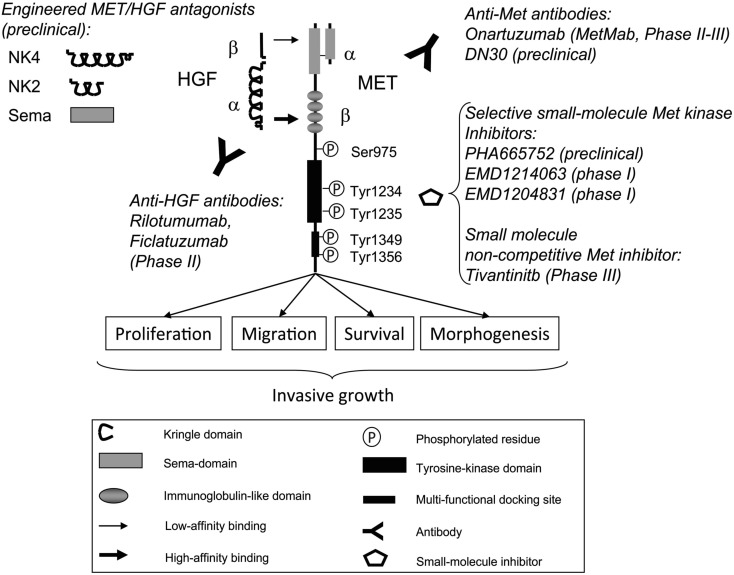
**Schematic representation of the receptor tyrosine kinase encoded by the MET oncogene, its ligand HGF, and the main experimental drugs against the receptor or the ligand currently under investigation (engineered MET/HGF antagonists, anti-HGF antibodies, anti-Met antibodies, small-molecule Met inhibitors)**. The α and β chains of both receptor and ligand are represented. In the receptor, the aminoacidic residues undergoing phosphorylation during signal activation are also indicated. For explanation of the specific domain functions, mechanisms of ligand-receptor interaction, and mechanism of action of experimental drugs, see text.

The MET proto-oncogene encodes the tyrosine kinase receptor for Hepatocyte Growth Factor (HGF), also known as Scatter Factor (SF; Giordano et al., 1989; Naldini et al., 1991). This ligand is synthesized as an inert single-chain precursor, and then converted into an active α- and β-chain heterodimer by extracellular proteases, including members of the blood coagulation system, such as urokinase-type plasminogen activator (uPA) and factor XII. Interestingly, HGF itself shares a high degree of homology with coagulation factors, as the α chain contains plasminogen-like “kringle” structural motifs, and the β chain contains a domain homologous to serine proteases, but devoid of enzymatic activity by substitution of critical aminoacids in the catalytic site (Figure 1; for a review see Trusolino and Comoglio, 2002).

The MET receptor is synthesized as a single-chain precursor that undergoes post-translational cleavage into two disulfide-linked α and β subunits. The extracellular domain contains two main regions, both involved in ligand binding. The first is the “sema domain,” named after its homology with the signaling molecules semaphorins, which includes the α chain and an N-terminal portion of the β chain. The second is the “immunoglobulin-like domain,” containing four disulfide-linked, loop structures (Figure 1; Gherardi et al., 2003). The intracellular domain of MET includes three functional portions with aminoacidic residues critical for modulation of signaling properties: (i) a juxtamembrane sequence including Ser^975^, which, upon phosphorylation, downregulates kinase activity; (ii) a catalytic region, containing Tyr^1234^ and Tyr^1235^, which, upon receptor dimerization and transphosphorylation, upregulate kinase activity; (iii) a carboxy-terminal sequence including Tyr^1349^ and Tyr^1356^ that works as a multifunctional docking site required and sufficient to recruit the core of cytoplasmic signal transducers and adaptors (Figure 1; for review see Trusolino and Comoglio, 2002; Trusolino et al., 2010).

Hepatocyte growth factor and MET control a complex biological program defined as “invasive growth” (Trusolino and Comoglio, 2002). This program coordinates cell proliferation with cell invasion, and provides protection from apoptosis usually occurring in cells removed from their physiological context. MET-driven invasive growth is a physiological program taking place during embryonic development and post-natal tissue growth and regeneration (Birchmeier and Gherardi, 1998; Boccaccio and Comoglio, 2006; Trusolino et al., 2010).

The signaling pathways that couple MET activation with the invasive growth biological program are largely shared with other growth factor receptors, and include in the first place MAP Kinase and PI-3 Kinase-AKT pathways, but also STAT3, p38, and NF-kB pathways. However, as compared with other growth factor receptors, MAP kinase and PI-3 Kinase are usually activated by MET with greater intensity and duration. The ability of MET to hyperactivate PI-3 kinase and MAPK signaling is thought to be essential for the pro-invasive activity. This hyperactivation results from aggregation of a complex signaling platform that amplifies the biochemical input, and translates it into specific biological outcomes. This platform includes the intracellular docking protein Gab1, and several cell surface proteins such as: (i) CD44v6, required to link the MET cytoplasmic tail to the actin cytoskeleton and, together with Gab1, to sustain activation of the MAP kinase cascade; (ii) α6β4 integrin, acting as a supplementary docking platform for amplification of PI-3 Kinase, MAP kinase, and SRC-dependent pathways; (iii) B family plexins, the receptors for semaphorins, which can trans-activate MET in the absence of HGF (for a review see Trusolino et al., 2010).

## The MET Oncogene in Cancer

Inappropriate activation of the MET oncogene has been reported in a wide variety of human tumors, where it supports execution of pathological invasive growth, leading to cancer aggressiveness and metastatic dissemination (Comoglio et al., 2008). MET genetic alterations are relatively rare, and include chromosomal rearrangements, a panel of point mutations, and gene amplification. The chromosomal rearrangement TPR-MET, observed in gastric cancers, results in the expression of a completely intracellular fusion protein that comprises a constitutive dimerization motif and the MET intracellular domain (Soman et al., 1991). Point mutations of MET were found in both hereditary and sporadic papillary renal cancer (Schmidt et al., 1997) and gastric carcinoma (Lee et al., 2000), in childhood liver carcinoma (Park et al., 1999), and lymph node metastases of head and neck squamous carcinoma (Di Renzo et al., 2000). Interestingly, MET point mutations have been recently associated with the so-called “cancers of unknown primary origin” (CUP). These are highly undifferentiated tumors that present at their very onset as metastatic dissemination in the absence of a detectable primary tumor (Stella et al., 2011). MET amplification, resulting in overexpression and constitutive kinase activation, has been found in gastric and esophageal carcinomas (Houldsworth et al., 1990), and medulloblastomas (Tong, 2004). Of special interests is MET amplification emerging in non-small cell lung carcinomas (NSCLC) treated with drugs targeting epidermal growth factor receptor (EGFR; Bean et al., 2007; Engelman et al., 2007). These results provided a rationale for combining EGFR and MET inhibitors in lung cancer clinical trials.

The most frequent cause of constitutive MET activation in human tumors is overexpression of a structurally normal protein, which results from transcriptional upregulation. Indeed, MET transcription can be sustained through mechanisms activated by frequent cancer genetic alterations such as Ras mutation (Ivan et al., 1997), p53 loss (Hwang et al., 2011), or MACC1 expression (Stein et al., 2009). In addition, MET transcription is induced by environmental cues, such as growth factors secreted by the adjacent stroma, including HGF itself, or by oxygen deficiency, or hypoxia, a frequent occurrence in the rapidly growing tumor tissue (Pennacchietti et al., 2003). Recently, we have shown that Met transcriptional upregulation, mediated by transcription factor NF-kB, and Met tyrosine kinase activation, leading to cell invasion and protection from apoptosis, occur when cells are exposed to ionizing radiations. Conversely, the use of Met inhibitors sensitizes cells to radiotherapy (De Bacco et al., 2011).

Finally, the discovery that MET protein translation is impaired by miRNA (Migliore et al., 2008), has led to the observation that loss of “tumor suppressor” miRNAs can cause MET overexpression during colorectal cancer progression (Migliore et al., 2012).

## Strategies to Target MET

MET inhibition has been pursued by trying to interfere with each of the main steps controlling MET activation, namely: (i) MET interaction with its ligand HGF; (ii) MET receptor oligomerization; (iii) MET catalytic activity; (iv) MET downstream signaling (for review see Comoglio et al., 2008; Gherardi et al., 2012). These studies have led to formulation of compounds that fall in the following three main classes.

### Engineered proteins antagonizing HGF and MET

The complex mechanism of interaction between HGF and MET has been elucidated through structural and crystallographic studies. HGF includes two binding sites for MET: a high-affinity binding site located in the α chain, which binds the MET immunoglobulin-like domains (Basilico et al., 2008), and a low-affinity binding site located in the β chain, which binds the MET sema domain (Stamos et al., 2004; Figure 1). Isolated domains of HGF or MET proteins have been shown to antagonize HGF-MET binding and block the ensuing MET biological effects in preclinical models. The best characterized antagonist is NK4, corresponding to the HGF α chain, and named after the “four kringle” (K) structural motifs (Figure 1). Interestingly, it has been observed that NK4 has a bifunctional role, targeting both the tumor and the microenvironment, as it displays also a powerful anti-angiogenic effect (for a review see Matsumoto and Nakamura, 2008). A similar effect has been reported also for an engineered molecule called “decoy MET,” corresponding to the isolated, soluble MET extracellular domain (Michieli et al., 2004). The anti-angiogenic effect of HGF/Met antagonists is explained by the ability of HGF to directly promote endothelial cell growth as well as VEGF expression (for a review see Gherardi et al., 2012). NK4, moreover, can exert an anti-angiogenic effect by mimicking angiostatin (for a review see Matsumoto and Nakamura, 2008). Finally, other engineered protein successfully tested in experimental models as HGF/Met antagonists are: (i) NK2, a naturally occurring splice variant of HGF, including the first two kringle domains (Chan et al., 1991), and (ii) the isolated Sema domain (see above; Kong-Beltran et al., 2004; Figure 1).

### Antibodies against HGF and MET

A humanized monoclonal antibody that binds the HGF β chain (rilotumumab, or AMG102, developed by Amgen; Figure 1), and inhibits HGF binding to MET, has displayed excellent neutralizing activity in experimental models (Burgess et al., 2006), and is currently tested in several Phase II clinical trials in recurrent glioblastoma, kidney, and gastric carcinoma, and other tumors^1^. Another humanized anti-HGF antibody, currently investigated in Phase II clinical trials for NSCLC, is ficlatuzumab (developed by AVEO; see text footnote 1).

The available antibodies against MET include DN30 (developed by Metheresis; Prat et al., 1998; Petrelli, 2006), and the so-called “METMab” (onartuzumab, developed by Genentech and Roche; Jin et al., 2008; Figure 1). DN30 is a monoclonal antibody that eventually induces proteolytic cleavage and release of the MET extracellular domain (see below). This process causes not only full receptor inhibition, but also ligand neutralization, which results from interaction of the solubilized MET extracellular domain with HGF bound to the extracellular matrix (“decoy effect”). However, on MET binding, DN30 retains a partial agonist activity that has been circumvented by transforming the original IgG divalent form into a monovalent form (Pacchiana, 2010). This antibody is in a preclinical development stage. METMab is a humanized monovalent antibody with neutralizing activity against MET, which, administered in association with EGFR inhibitors to patients affected by NSCLC, has significantly increased the “progression-free survival” period of patients that expressed high levels of MET (Spigel, 2011).

### Small molecule MET inhibitors

MET catalytic activity can be blocked by small molecule inhibitors that compete for ATP binding at the kinase active site. A group of these compounds, sharing a common structure (an indolin-2-one core), display specific activity against MET, and include, among the most selective and potent, PHA665752 (Pfizer; Smolen, 2006), EMD1214063, and EMD1204831 (Serono), and JNJ38877605 (Jhonson and Jhonson; De Bacco et al., 2011; Figure 1). These molecules are still in a preclinical or early clinical developmental phase, and, in the case of JNJ38877605, have raised serious concerns for possible human toxicity (for a review see Peters and Adjei, 2012). Another small molecule MET inhibitor, Tivantinib (also known as ARQ197, developed by Arqule), does not compete for ATP binding to the MET catalytic site, and acts through alternative but still poorly characterized mechanisms (Eathiraj et al., 2011; Figure 1). However, it is in advanced stage of clinical development and, in combination with EGFR inhibitors, has shown remarkable activity in extending the progression-free survival of patients with NSCLC (Sequist et al., 2011).

Other inhibitors with broader specificity, concomitantly targeting the tyrosine kinase activity of MET and other receptors, are currently tested in Phase II-III trials. Among the most investigated, Crizotinib (also known as PF-02341066, developed by Pfizer) inhibits MET, ALK, RON, AXL, and TIE2. In phase III trials, Crizotinib has shown a striking activity in NSCLC patients harboring the rare EML4-ALK translocation, leading to expression of a constitutively active ALK kinase (Kwak et al., 2010). Cabozantinib (also known as XL184, developed by Exelixis) inhibits MET, VEGFR2, RET, KIT, FLT3, and TIE2. This compound showed significant activity against a variety of primary and metastatic tumors, among which the most remarkable are metastatic castration-resistant prostate cancer (Hussain, 2011), and medullary thyroid cancer (Kurzrock et al., 2011). Obviously, in case of broad specificity inhibitors, it is hard to discriminate the contribution of MET inhibition to the overall therapeutical effect.

## MET Regulation by Proteases and Its Therapeutic Implications

Over the past few years the existence of a cross-talk between proteases and tyrosine kinases became increasingly evident (Lopez-Otin and Hunter, 2010). Earlier studies described the ability of MET to activate several proteases, such as Matrix-metalloproteinase-9 (MMP-9; Harvey et al., 2000). The importance of the reverse regulation, i.e., modulation of MET (or other receptor tyrosine kinases) by proteases, became apparent only recently, but proved to be relevant for the development of new cancer therapies (Schelter, 2010; Schelter et al., 2011).

One of the first findings indicating regulation of proteases by MET was that co-expression of MET and HGF increased the metastatic potential of NIH3T3 fibroblasts *in vivo*, by sustaining constitutive activation of MET signaling and proteolytic activity (Rong et al., 1994). As proteolytic activity is generally considered a prerequisite for metastasis (Deryugina and Quigley, 2006; Fingleton, 2006), several subsequent studies addressed the regulatory effect of MET signaling on pro-invasive proteases. The observation that pro-invasive MMP-9 can be induced by MET signaling (Harvey et al., 2000) is of special interest, as MMP-9, as well as its close relative MMP-2, can cleave collagen type IV, the main component of the basement membrane, a physiological boundary that only fully malignant cells can trespass (Egeblad and Werb, 2002). Beside activation of members of the matrix-metalloproteinase family, it was also shown that MET signaling induces expression and activity of other proteases, including urokinase-type plasminogen activator (uPA) (Jeffers et al., 1996). Taken together, these findings significantly contributed to provide a mechanistic explanation for the pro-invasive effect of HGF, or “invasive growth” (Trusolino and Comoglio, 2002).

It is well known that termination of MET signaling is achieved by down-regulation of this receptor, which occurs after induction of phosphorylation. The canonical process of MET down-regulation mostly relies on endocytosis, and seems to be protease-independent (for a review see Trusolino et al., 2010). However, there are parallel mechanisms of down-regulation, which are indeed protease-dependent. One mechanism was found in apoptotic cells, where MET is cleaved by caspases, resulting in blockage of MET-mediated survival signals (Foveau et al., 2007). This process could be inactivated in cancer cells, which are often unable to unleash the apoptotic pathway, leading to MET accumulation, and further support of anti-apoptotic signals.

A second mechanism of protease-dependent MET down-regulation involves extracellular proteases. We previously reported that, in a murine model, elevated levels of systemic Tissue Inhibitor of Metalloprotease-1 (TIMP-1) promoted metastatic dissemination of tumor cells to the liver. This effect was dependent on increased activity of the MET signaling pathway in the liver tissue microenvironment (Kopitz et al., 2007). This observation suggested the hypothesis that the endogenous broad spectrum inhibitor TIMP-1 inhibited a potential MET sheddase, causing accumulation of MET on the cell surface, and hyperactivation of MET signaling (Kopitz et al., 2007). Earlier reports (Nath et al., 2001; Lee et al., 2002) had shown that, under physiological conditions, MET shedding was mediated by a protease sensitive to tissue inhibitor of metalloproteinases-3 (TIMP-3), thus likely belonging to “A Disintegrin And Metalloproteinase (ADAM)” family, which includes prominent mediators of cell surface protein shedding (Murphy, 2008). In particular, it was known that TIMP-3 can inhibit ADAM-10 and ADAM-17, whereas TIMP-1 can only inhibit ADAM-10 (Amour et al., 1998, 2000). In our attempt to explain the pro-metastatic effect of TIMP-1, mediated by MET accumulation, we obtained the first evidence that ADAM-10 is a MET sheddase (Kopitz et al., 2007), a finding further confirmed by other studies (Schirrmeister et al., 2009; Schelter, 2010; Doberstein et al., 2011; Schelter et al., 2011). Moreover, also ADAM-17, a close functional homolog of ADAM-10, was identified as a potential MET sheddase (Foveau et al., 2009). Taken together, these findings explain the early observation that MET shedding can rely on TIMP-3-sensitive proteases, as both ADAM-10 and ADAM-17 are inhibited by TIMP-3 (Amour et al., 1998, 2000). We concluded that, in the liver microenvironment, elevated levels of TIMP-1 inhibited MET shedding in liver cells, leading to MET accumulation and increased signaling, thereby providing a fertile soil for colonization by metastatic cells (host effect; Kopitz et al., 2007). Moreover, we showed that inhibition of MET shedding by TIMP-1 occurs also in cancer cells, thus sustaining their invasive growth potential and ability to colonize the liver (Schelter et al., 2011).

Taken together, these studies show that regulation of MET signaling by proteases is relevant in the context of cancer. Therefore, the question arises whether this mechanism can be exploited to develop anti-cancer therapies. Previously, it was shown that a MET-specific monoclonal antibody (DN30) induces shedding of MET and inhibition of MET signaling (Petrelli, 2006), strongly suggesting the involvement of an ADAM protease in the antibody’s mechanism of action. Indeed we recently showed that ADAM-10 (but not ADAM-17) mediated MET shedding induced by DN30, and thus it was critical for the therapeutic effect of this antibody (Schelter, 2010). Knockdown of ADAM-10, but not of ADAM-17, abolished MET down-regulation in different tumor cell lines, and compromised the DN30 ability to block MET signaling (Schelter, 2010). Moreover, also the DN30 ability to inhibit HGF-dependent tumor cell scattering and invasiveness *in vitro* was shown to depend on ADAM-10 (Schelter, 2010). These findings led to the conclusion that patients should be screened for ADAM-10 expression before being treated with DN30, as ADAM-10 is required for the activity of this antibody. Furthermore, these observations suggest the possibility to finely tune the specificity of targeted therapies, such as the DN30 antibody, by combining the activity of the antibody and the protease required for its activity.

## Conclusion

The tyrosine kinase receptor encoded by the MET oncogene is expressed in a wide variety of tumors, where it often displays a deregulated activity that leads to pathological “invasive growth,” featuring invasion, and metastasis. Moreover, MET is expressed by endothelial cells, and can be involved in tumor angiogenesis. The connection between MET and tumor microenvironment is emphasized by the interplay with matrix proteases, such as ADAM-10, that modulate its activity. Overall, MET hyperactivation is likely to play a crucial role in tumor onset and progression. MET and its ligand HGF are attractive pharmacological targets: the ligand and the receptor extracellular domain can be blocked by antibodies, or by engineered protein antagonists, while the tyrosine kinase activity can be inhibited by small molecules. Antibodies and kinase inhibitors are currently tested in clinical trials with encouraging results.

## Conflict of Interest Statement

The authors declare that the research was conducted in the absence of any commercial or financial relationships that could be construed as a potential conflict of interest.

## Footnotes

^1^http://www.vai.org/metclinicaltrials/
